# *Forsythia suspensa* Suppresses House Dust Mite Extract-Induced Atopic Dermatitis in NC/Nga Mice

**DOI:** 10.1371/journal.pone.0167687

**Published:** 2016-12-09

**Authors:** Yoon-Young Sung, Taesook Yoon, Seol Jang, Ho Kyoung Kim

**Affiliations:** 1 Mibyeong Research Center, Korea Institute of Oriental Medicine, Daejeon, Republic of Korea; 2 College of Pharmacy, Graduate School of Pharmaceutical Sciences, Ewha Womans University, Seoul, Republic of Korea; Purdue University, UNITED STATES

## Abstract

*Forsythia suspensa* (*F*. *suspensa*) is a traditional medicine for treatment of inflammation. In this study, we evaluated the therapeutic effects of an ethanol extract from *F*. *suspensa* fruits on atopic dermatitis both *in vivo* and *in vitro*. We investigated the inhibitory effects of *F*. *suspensa* extract on the development of atopic dermatitis-like skin lesions in an NC/Nga mouse model exposed to *Dermatophagoides farinae* crude extract. Topical application of *F*. *suspensa* extract to the mice attenuated the atopic dermatitis symptoms, including increased dermatitis severity score, ear thickness, infiltration of inflammatory cells in the skin lesions, serum levels of IgE, TNF-α, and histamine, and expression of chemokines, cytokines, and adhesion molecules in ear tissue. In addition, *F*. *suspensa* extract inhibited the production of chemokines in TNF-α/IFN-γ-activated human keratinocytes. High-performance liquid chromatography analysis of FSE revealed the presence of four chemical constituents (forsythiaside, phillyrin, pinoresinol, and phylligenin). These compounds inhibited the production of chemokines in TNF-α/IFN-γ-activated human keratinocytes. These results suggest that the *F*. *suspensa* might be a useful candidate for treating allergic skin inflammatory disorders.

## Introduction

Atopic dermatitis (AD) is a chronic inflammatory skin disorder. It is characterized by increased serum immunoglobulin (Ig) E levels, intense pruritus, and cutaneous hypersensitivity to environmental triggers [1]. The skin-infiltrating inflammatory cells containing mast cells, eosinophils, Langerhans cells, and CD4+ lymphocytes expressing skin colonization antigen (CLA) play a crucial role in the initiation and exacerbation of inflammation in AD [2]. T-helper (Th) 2 lymphocytes, which produce interleukin (IL)-4, IL-5, and IL-13, play major roles in the pathogenesis of AD in the early stage [3]. Th1 lymphocytes, which mainly produce pro-inflammatory cytokines such as tumor necrosis factor alpha (TNF-α) and interferon gamma (IFN-γ), contribute to pathogenesis of AD during the chronic stage [4]. The keratinocyte activation is a hallmark of the development of AD in acute and chronic phases [5]. In the skin, keratinocytes exhibit an exaggerated production of chemokines and cytokines and participate in induction and maintenance of inflammation [6]. Many chemokines and their receptors affect cell trafficking and infiltration of the inflammatory cells by lymphocyte chemotaxis to the skin [2]. Thymus- and activation-regulated chemokine/chemokine (C-C motif) ligand (CCL) 17 (TARC/CCL17), macrophage-derived chemokine (MDC/CCL22), regulated on activation, normal T cell expressed and secreted (RANTES/CCL5), monocyte chemotactic protein-1 (MCP-1/CCL2), eotaxin/CCL11, MCP-4/CCL13, and eotaxin 3/CCL26 affect the migration of the T lymphocytes, dendritic cells, monocytes, and eosinophils [7]. Adhesion molecules, such as intercellular adhesion molecule (ICAM)-1 and vascular adhesion molecule (VCAM)-1, are proteins on the cell surface that are involved in the interactions between lymphocytes and antigen-presenting cells in inflammatory skin diseases and have significant roles in the immune and inflammatory mechanisms [8].

*Forsythia suspensa* (Thunb.) Vahl. (*F*. *suspensa*) is a flowering plant species found in Korea, China, Japan, and the many European countries [9]. The fruit of this plant has been used as a traditional medicine for inflammatory disease [9, 10]. It has also been reported that *F*. *suspensa* inhibits carrageenan-induced edema and acetic acid-mediated induction of vascular permeability in mouse model [11]. *F*. *suspensa* extract (FSE) inhibits 5-lipoxygenase as a therapeutic target enzyme for dermatological disorders such as psoriasis [12]. In addition, it has been reported that *F*. *suspensa* inhibits mast cell-mediated allergic inflammatory reactions. In this study, *F*. *suspensa* was shown to have inhibitory effects on histamine release and paw edema induced compound 48/80 as well as vascular permeability in rat peritoneal mast cell and mice [13]. However, the effects of *F*. *suspensa* on AD have not yet been investigated. Therefore, this study was designed to elucidate the effect of *F*. *suspensa* on AD-like skin lesions and its underlying mechanisms. We evaluated the inhibitory activities of (FSE) on allergic inflammation using an NC/Nga mouse AD model exposed to house dust mites and a human keratinocytes (HaCaT) cell model. Repeated exposure of NC/Nga mice to *Dermatophagoides farinae* (*D*. *farinae*) crude extract (DfE), which is a common environmental mite allergen on humans, induced AD-like skin lesions under specific pathogen-free conditions [14]. The effects of FSE were compared with those of tacrolimus, an immunosuppressant commonly used to AD [15–17]. Although the efficacy is weaker than topical glucocorticoids, tacrolimus ointment can be used as a rapidly effective and safe treatment for the management of AD [18–19].

## Materials and Methods

### Animals

Specific pathogen-free 8-week-old male NC/Nga mice were purchased from Central Lab Animal Inc. (Seoul, Korea) and maintained for 1 week prior to experiment. The mice were housed individually in ventilated cages of an animal room under controlled environmental condition (12-h light/dark cycle, 22±1°C temperature, 50±10% relative humidity). Corncob natural bedding material (Premium grade Corn Cob, Nepco, Warrensburg, NY, USA) to control ammonia levels was used with these cages. Mice were provided with a standard laboratory diet (LabDiet 5L79, Orient, Sungnam, Korea) and water *ad libitum* in the specific pathogen-free facility (KIOM Laboratory animal research center, Daejeon, Korea).

### Ethics statement

The experiments were approved by the Institutional Animal Care and Use Committee of the Korea Institute of Oriental Medicine (Permit No. 10–162) and all procedures were performed in accordance with the Guide for the Care and Use of Laboratory Animals of the National Institutes of Health (NIH publication No. 85–23, revised 1996). All surgery was performed under pentobarbital anesthesia and approved by the Institutional Animal Care and Use Committee of the Korea Institute of Oriental Medicine. All efforts were made to minimize suffering of mice.

The aim of this study is to investigate the therapeutic effects of FSE on DfE-induced atopic dermatitis mice. Thus, for comparison between the two groups, these animal were not administered any ointments to decrease the pain and/or itching. However, to minimize potential pain and distress during the experiment, AD-like skin lesions were induced in mice by treatment with a small amount of DfE ointment within a short period of time. The clinical symptoms recovered slowly in DfE-treated control mice after last DfE application on the 21st day. Environmental and behavioral factors affect the emotional component of pain perception in animals. The mice were gently handled to minimize the animal’s discomfort. Also, the mice were singly housed in cages with a deep bedding material.

### Preparation of *Forsythia suspensa* extract

*F*. *suspensa* dried fruits were purchased from an oriental drug store (Omniherb Co., Yeoungcheon, Korea). A voucher specimen (No. KIOM-78039) was deposited at the herbarium of the Department of Herbal Resources Research of KIOM (Daejeon, Korea). The dried plants (50 g) were extracted three times in 0.5 L of 70% ethanol for 60 min using an ultrasonic bath (model 8510, Branson Co., Danbury, CT, USA). The ethanol supernatants were filtered and evaporated *in vacuo* to yield the 70% ethanol extract (5.42 g). The extract yield was 10.84%. For *in vitro* experiments, the ethanol extract powder was dissolved in phosphate-buffered saline (PBS).

### Experimental animal model of atopic dermatitis and *Forsythia suspensa* extract application

AD-like skin lesions were induced in NC/Nga mice by treatment with DfE as described previously [20]. To induce AD, the shaved dorsal skin was treated with 100 mg of ointment containing crude DfE (Biostir Inc., Kobe, Japan) two times a week for 3 weeks. Mice were anesthetized using pentobarbital, and the back hair of each mouse was shaved with a clipper 1 day before the experiments. The skin dermal barrier was disrupted by applying sodium dodecyl sulfate (4% SDS, 150 μL) on the shaved back skin 3 h before the DfE application. Thus, SDS was applied two times a week for 3 weeks. From the 11th day after the first DfE application, FSE was applied every day for 23 days. Mice were randomly assigned into four groups (n = 7 animals in each group): untreated normal group, DfE-treated control mice (100 mg/mouse), DfE-treated mice that applied 400 μg of FSE, and DfE-treated mice that applied 100 μg of tacrolimus (0.1% Protopic ointment, Astellas Pharma, Deerfield, IL, USA). Tacrolimus was used as a positive control. For topical application, the FSE powder was dissolved in acetone:olive oil [4:1 (v/v)] solution. In normal and DfE-treated control mice, the same volume of vehicle was applied instead of FSE. Thickness of each ear was measured twice a week. Throughout the experimental period, the body weights were measured weekly and physical conditions of the mice were monitored daily. Mice were sacrificed by anesthesia with pentobarbital (intraperitoneally, 100 mg/kg) on the 34th day after the first treatment of DfE. At the autopsy, blood was collected form the posterior *vena cava*, and the skin samples from ear and back were excised for further analysis.

### Evaluation of skin lesion severity

The lesions on the ear and back skin were assessed macroscopically according to the following four symptoms: erythema/hemorrhage, edema, scarring/dryness, and excoriation/erosion, and the total clinical dermatitis severity score for each mouse was defined as the sum of the individual scores (0, no symptoms; 1, mild; 2, moderate; 3, severe), ranging from 0 to 12 [21]. At the start of this experiment, the score for each group was 0. Then, the dermatitis severities on the skin lesions were scored twice a week. These visual assessments were performed by at least two independent investigators. The changes in the skin symptoms of the NC/Nga mice were evaluated by viewing photographs of the mice.

### Serum histamine, TNF-α, and total IgE levels measured by ELISA

Blood samples were collected from the mice after sacrifice and centrifuged at 2,000 x *g* for 20 min at 4°C. Serum was then collected and stored at -70°C for further investigations. Histamine levels in serum were quantified using a histamine enzyme immunoassay kit (Oxford Biomedical Research Inc., Oxford, MI, USA) according to the manufacturer’s instructions. Total IgE and TNF-α concentrations were quantified using a mouse IgE enzyme-linked immunosorbent assay (ELISA) kit (Shibayagi, Gunma, Japan) and a mouse TNF-α ELISA kit (R&D systems Inc., Minneapolis, MN, USA), respectively.

### Reverse transcriptase-polymerase chain reaction (RT-PCR)

Total RNA from the ear tissues was isolated using the easy-BLUE total RNA extraction kit (Intron, Seoul, Korea). The complementary DNA (cDNA) was synthesized from 1 μg of total RNA with Maxime RT premix (Intron, Seoul, Korea) containing dNTPs, oligo-dT primer, optiscript reverse transcriptase, and water. The reverse transcription was performed at 45°C for 60 min and the cDNA synthesis reaction was inactivated at 95°C for 5 min. The cDNA was then amplified by a Taq PCR master mix kit (Qiagen, Tokyo, Japan). Sequences of gene-specific primers were designed using primer 3 software and GenBank database (Table 1). The 1 μL of cDNA and 10 μL of a 2 x Taq PCR master mix (Qiagen, Tokyo, Japan) containing 1.5 mM MgCl_2_, 0.1 μM of each primer, and water were mixed together in a final 20-μL amplification mixture and pre-incubated at 94°C for 15 min. PCR amplification was performed for 35 cycles (denaturation at 94°C for 30 s, annealing at 60°C for 30 s, and extension at 72°C for 1 min). Quantitative analysis of PCR bands was performed with the NIH Image J software program. The expression levels of interest genes were normalized using glyceraldehyde-3-phosphate dehydrogenase (GAPDH).

**Table 1:** Primer sequence used for RT-PCR.

Genes	Sense	Antisense	Accession number	Length (bp)
TARC	CAGGAAGTTGGTGAGCTGGTATA	TTGTGTTCGCCTGTAGTGCATA	NM_011332	300
RANTES	GCTCCAATCTTGCAGTCGTGTT	ATTTCTTGGGTTTCGTGGTCG	NM_013653	283
MDC	TCTGATGCAGGTCCCTATGGT	TTATGGAGTAGCTTCTTCAC	NM_009137	207
IL-4	TCAACCCCCAGCTAGTTGTCA	CATCGAAAAGCCCGAAAGAG	NM_021283	313
TNF-α	CCTGTAGCCCACGTCGTAGC	TTGACCTCAGCGCTGAGTTG	NM_013693	373
ICAM-1	CCTCTGCTCCTGGCCCTGGT	CGGACTGCTGTCCTCCCCGA	NM_010492	237
VCAM-1	TCGCGGTCTTGGGAGCCTCA	TCGCGGTCTTGGGAGCCTCA	MM_011693	213
GAPDH	AAGCTGTGGCGTGATGGCCG	TGGGCCCTCAGATGCCTGCT	NM_008084	228

Abbreviations: GAPDH, glyceraldehyde-3-phosphate dehydrogenase; ICAM, intercellular adhesion molecule; IL, interleukin; MDC, macrophage-derived chemokine; RANTES, regulated on activation, normal T cell expressed and secreted; TARC, thymus- and activation-regulated chemokine; TNF-α, tumor necrosis factor alpha; VCAM, vascular adhesion molecule.

### Histological analysis

Tissue samples (5 x 5 mm area) from the ear and dorsal skin of mice (n = 7) were removed with scissors 24 h after final FSE treatment, fixed in 10% formalin for 16 h, and embedded in paraffin. Then, 2–3 μm sections were stained with hematoxylin and eosin solution (Sigma-Aldrich, St. Lowis, MO USA). Histopathology was scored as follows: no lesion, 0; minimal, 1; mild, 2; moderate, 3; and severe, 4. The skin sections were also stained with toluidine blue for investigating the number of mast cells. Histopathological changes and number of mast cells were examined under light microscopy (Olympus CX31/BX51, Olympus, Tokyo, Japan). Total number of the mast cells or eosinophils in five random sites (x200) in each specimen (n = 7) was counted under a microscope, and the mean number of cells in one site was calculated.

### Cell culture and reagents

Human HaCaT (immortalized keratinocyte) cells were obtained from Dr. H. K. Shin. (KIOM, Daejeon, Korea). Dulbecco’s Modified Eagle’s Medium (DMEM), fetal bovine serum (FBS), 100 U/mL penicillin, and 100 μg/mL streptomycin were obtained from Gibco BRL (Grand Island, NY, USA). TNF-α and IFN-γ were obtained from R&D Systems (Minneapolis, MN, USA). The HaCaT cells were cultured in DMEM supplemented with 10% FBS, 100 U/mL penicillin, and 100 μg/mL streptomycin at 37°C. Forsythiaside, phillyrin, pinoresinol, and phylligenin (Purity: 98%) were purchased from Chengdu Biopurify Phytochemicals (Chengdu, China).

### Cell viability assay

The effects of FSE on HaCaT cell viability was evaluated using a 3-(4,5-dimethylthiazol-2-yl)-2,5-diphenyltetrazolium bromide (MTT) colorimetric assay [22]. HaCaT cells (5 x 10^4^ cells/well) were seeded into each well of 96-well plates and incubated with various concentrations (25, 50, 100, 200, and 400 μg/mL) of FSE or compounds (1, 2, 5, and 10 μg/mL) for 24 h. Then, 0.5 mg/mL MTT solution (100 μL) was added into each well. After incubation for 2 h at 37°C, Dimethyl sulfoxide was added to solubilize the purple formazan crystals. The absorbance at 540 nm was measured with a microplate reader (BioRad, Hercules, CA, USA).

### Measurement of chemokine production by ELISA

HaCaT cells (3 x 10^5^ cells/well) were seeded into each well of 24-well plates, and stimulated with TNF-α (10 ng/mL) and IFN-γ (10 ng/mL). The cells were subsequently incubated with FSE (25, 50, 100, 200, and 400 μg/mL) or compounds (1, 2, 5, and 10 μg/mL) for 24 h. As positive control, cells were treated with dexamethasone (10^−4^ M). The production of RANTES, TARC, and MDC from the supernatant was measured using a human RANTES, TARC, and MDC ELISA kit (R&D Systems Inc., Minneapolis, MN, USA). Anti-human captured antibody was treated to each well of a 96-well plate, and the plate was then blocked with 1% bovine serum albumin at room temperature. Supernatants were added in the wells, and the plate was incubated for 2 h. After washing 5 times, anti-human detection antibody was added. After further incubation for 2 h at room temperature, wells were washed five times and streptavidin-horseradish peroxide conjugate was treated, and the plate was then incubated for 20 min. After washing 7 times with washing buffer, the enzyme reaction was initiated by adding 100 μL of substrate and the reaction then was allowed to continue for 20 min. Finally, the reaction was finished by adding 50 μL of stop solution to each well. Absorbance at 450 nm was determined using a microplate reader (BioRad, Hercules, CA, USA).

### Quantitative high-performance liquid chromatography (HPLC) analysis

The sample was analyzed by reverse phase-high performance liquid chromatography using Waters e2695 liquid chromatography system (Waters Co., Milford, MA, USA), equipped with a Waters 2998 photodiode array detector. Data processing was carried out with the Empower software (Waters Co.). The Phenomenex Luna C18 column (250 mm x 4.6 mm; particle size 5 μm; Phenomenex, Torrance, CA, USA) was used as the stationary phase and 0.3% (v/v) acetic acid aqueous solution (A) and methanol (B) were used as the mobile phase. The elution conditions involved holding the starting mobile phase at 70% A and 30% B and applying a gradient of 10% A and 90% B for 40 min. A wash with 100% B was applied for 10 min, followed by equilibration at 70% A and 30% B for 10 min. The flow rate was 1.0 mL/min and the injection volume for all the samples was 10 μL. Peaks were identified by comparing retention times and UV spectra with those of commercial standards. Components were quantified based on peak areas at 235 nm. Quantitation was performed based on a mixture of external standards of known concentration, which were analyzed in duplicate before and after analyzing the samples. Peak areas were used to calculate the quantities of compounds in the samples. The calibration curves of the standards ranging from 11.9 to 336 μg/mL (five levels) revealed good linearity, with R2 values exceeding 0.99 (peak area vs. concentration). HPLC-grade reagents, methanol and water were obtained from J. T. Baker (Phillipsburg, NJ, USA).

### Statistics

Statistical significance was analyzed using one-way analysis of variance (ANOVA) followed by Tukey’s multiple comparison test to compare differences among groups (*p* < 0.05). All data were expressed as the mean ± standard error of the mean (S.E.M).

## Results

### *Forsythia suspensa* extract suppresses DfE-induced AD-like skin lesions in NC/Nga mice

**Fig 1:**
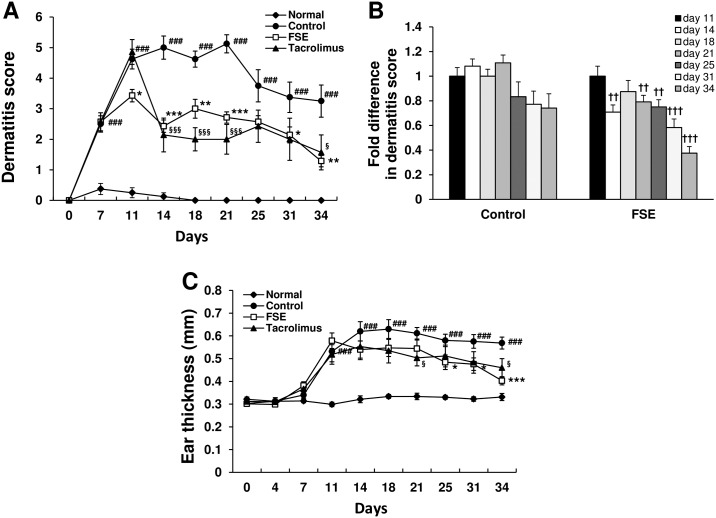
FSE suppresses *Dermatophagoides farinae* extract (DfE)-induced atopic dermatitis in NC/Nga mice. (A) Dermatitis severity score. (B) Fold difference in dermatitis score. Dermatitis score at the different time points was compared to the highest dermatitis score (day 11) within a group. (C) Ear thickness. DfE was applied to the shaved back skin and both surfaces of each ear twice per week for 3 weeks. Results are expressed as mean ± S.E.M for 7 mice. Normal, untreated group; Control, DfE-treated group; FSE, FSE-treated group. ^###^ *p* < 0.001 versus Normal; * *p* < 0.05, ** *p* < 0.01, and *** *p* < 0.001 FSE versus Control; ^§^ *p* < 0.05, ^§§^ *p* < 0.01, and ^§§§^ *p* < 0.001 Tacrolimus versus Control. ^††^*p* < 0.01, and ^†††^ *p* < 0.001 Dermatitis score at the different time points versus the highest dermatitis score in the FSE-treated group.

We investigated whether FSE prevents AD-like skin lesions in a mouse model. During the AD induction period, we measured ear swelling and determined a dermatitis severity score. In DfE-treated control mice, clinical symptoms such as skin dryness, edema, scarring, erythema, hemorrhage, erosion, and excoriation were observed. As shown in Fig 1A, the clinical dermatitis severity scores increased gradually with the DfE treatment and these AD symptoms continued until day 34. In contrast, the dermatitis severity scores in FSE- and tacrolimus-treated mice were lower than in DfE-treated control mice (Fig 1A). Compared to the highest dermatitis score (day 11) for the FSE group, the changes at subsequent time points were statistically significant (Fig 1B, *p* < 0.01). Ear thickness increased gradually in DfE-treated control mice, and the treatment of FSE and tacrolimus suppressed this DfE-induced ear swelling after 4 weeks of first AD induction (day 34, Fig 1C). Treatment with FSE significantly suppressed DfE-induced ear swelling on day 25 and these changes at subsequent time points continued. Throughout the experimental period, the body weights were measured weekly and physical conditions of the mice were monitored every day. Body weight in NC/Nga mice during the experiment revealed no significant differences among the groups. DfE-treated NC/Nga mice showed the mild itching behavior. None of the mice became severely ill or died prior to the experimental endpoint.

**Fig 2:**
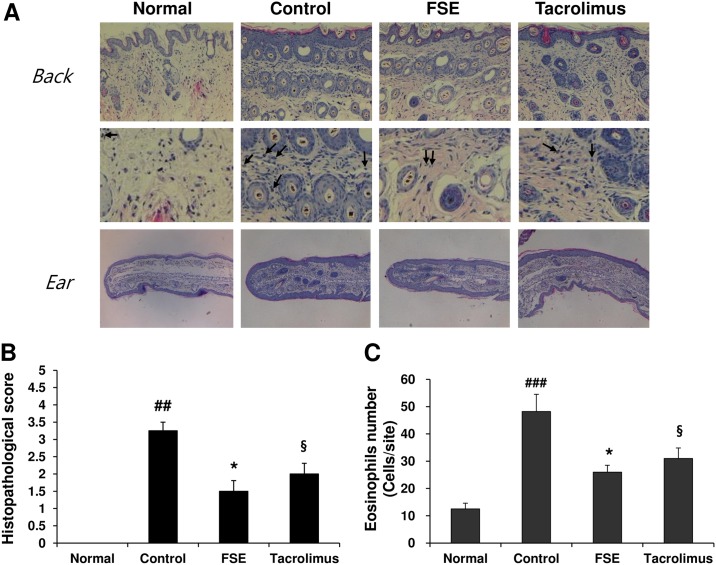
FSE suppresses tissue inflammation and infiltration of immune cells in NC/Nga mice. Back skin and ear sections were stained with (A) hematoxylin and eosin (H&E), and (B) histopathology was scored as described in Section 2 (original magnification x200). The arrows indicate the H&E-stained eosinophils. (C) Eosinophils were counted at five randomly selected sites of hematoxylin and eosin-stained sections. Results are expressed as mean ± S.E.M for 7 mice. ^##^ *p* < 0.01 and ^###^*p* < 0.001 versus Normal; * *p* < 0.05, ** *p* < 0.01, and *** *p* < 0.001 FSE versus Control; ^§^ *p* < 0.05, ^§§^ *p* < 0.01, and ^§§§^ *p* < 0.001 Tacrolimus versus Control.

Treatment with FSE markedly reduced inflammatory cell infiltration, hyperkeratosis (thickening of the epidermis), and ulcers in ear and back skin lesions (Fig 2A and 2B). Furthermore, FSE significantly reduced the number of eosinophils in the back skin lesions of DfE-treated NC/Nga mice (Fig 2C).

**Fig 3:**
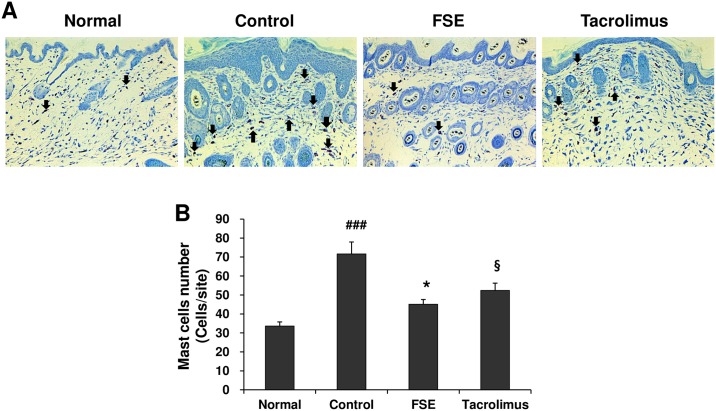
FSE reduces infiltration of mast cell in the back skin lesions of NC/Nga mice. (A) The back skin lesions were stained toluidine blue and (B) the number of mast cells in toluidine blue-stained sections were counted (x200). Mast cells were counted at five randomly selected sites of toluidine blue-stained sections. Results are expressed as mean ± S.E.M for 7 mice. ^##^ *p* < 0.01 and ^###^ *p* < 0.001 versus Normal; * *p* < 0.05, ** *p* < 0.01, and *** *p* < 0.001 FSE versus Control; ^§^ *p* < 0.05, ^§§^ *p* < 0.01, and ^§§§^ *p* < 0.001 Tacrolimus versus Control.

**Fig 4:**
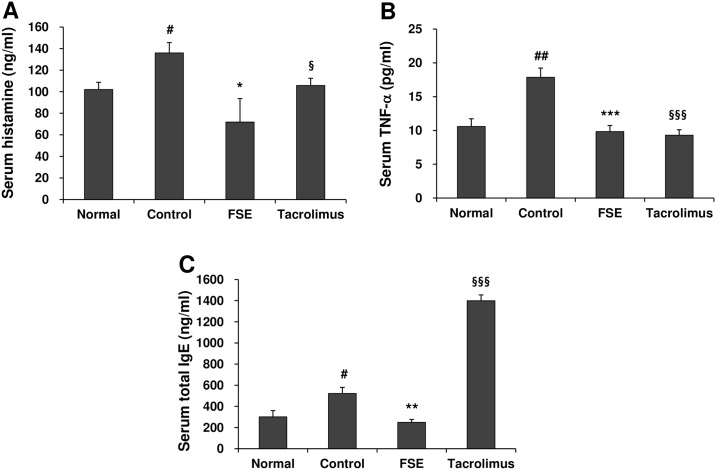
FSE reduces the serum levels of histamine, TNF-α, and total IgE in NC/Nga mice. The concentrations of (A) histamine, (B) TNF-α, and (C) total IgE. Results are expressed as mean ± S.E.M for 7 mice. ^#^ *p* < 0.05 and ^##^ *p* < 0.01 versus Normal; * *p* < 0.05, ** *p* < 0.01, and *** *p* < 0.001 FSE versus Control; ^§^ *p* < 0.05, ^§§^ *p* < 0.01, and ^§§§^ *p* < 0.001 Tacrolimus versus Control.

In the development of AD, mast cell-secreted histamine, cytokines, and various inflammatory mediators contribute to the allergic inflammatory response [23]. An increased level of serum IgE is the most characteristic feature in allergic AD [24]. Therefore, we examined the infiltration of mast cells in the AD skin lesions and the levels of histamine, IgE, and TNF-α in the serum of mice. FSE decreased the infiltration of mast cell in the AD skin lesions (Fig 3A and 3B) and serum levels of histamine and TNF- (Fig 4A and 4B). Compared with the AD mice, total serum IgE levels was markedly reduced in the FSE-treated mice (Fig 4C). These results suggest that FSE suppresses DfE-induced AD-like skin lesions in NC/Nga mice.

### FSE suppresses the expression of inflammatory mediators in ear tissues

**Fig 5:**
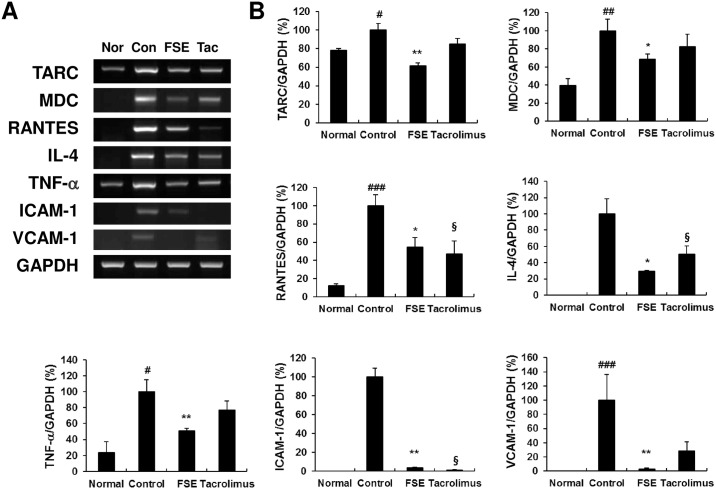
FSE inhibits the expression of chemokines, inflammatory cytokines, and adhesion molecules in the ear tissue of NC/Nga mice. (A) The mRNA expression of TARC, MDC, RANTES, IL-4, TNF-α, ICAM-1, and VCAM-1. (B) Quantitative analysis of PCR bands was performed with the NIH ImageJ program. Results are expressed as mean ± S.E.M for 7 mice. ^#^ *p* < 0.05, ^##^ *p* < 0.01, and ^###^ *p* < 0.001 versus Normal; * *p* < 0.05, ** *p* < 0.01, and *** *p* < 0.001 FSE versus Control; ^§^ p < 0.05, ^§§^ p < 0.01, and ^§§§^ p < 0.001 Tacrolimus versus Control.

We investigated the effect of FSE on the mRNA expression of inflammatory cytokines, chemokines, and adhesion molecules in the ear tissue. All inflammatory mediators increased in the DfE-induced AD control mice, and FSE suppressed the expression of chemokines (TARC, MDC, and RANTES), Th1/Th2 cytokines (TNF-α and IL-4), and adhesion molecules (ICAM-1 and VCAM-1) (Fig 5B). These results suggest that FSE suppresses the expression of inflammatory mediators, leading to inhibition of AD caused by the infiltration inflammatory cells.

### FSE inhibits the production of chemokines in human keratinocytes

**Fig 6:**
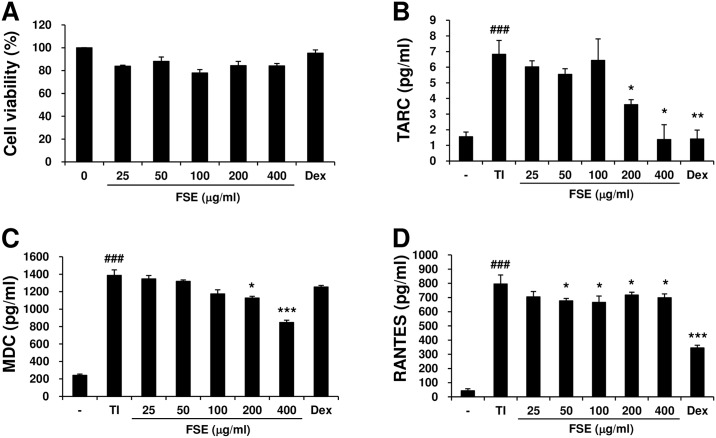
FSE inhibits the production of chemokines in TNF-α-and IFN-γ-treated human keratinocytes. HaCaT cells (3 x 10^5^ cells/well) were seeded into each well of 96-well plates and stimulated with or without FSE (25, 50, 100, 200, and 400 μg/mL) for 24 h. (A) Cell viability was measured using the MTT assay. Production levels of TNF-α-and IFN-γ-induced (B) TARC, (C) MDC, and (D) RANTES chemokines. HaCaT cells (3 x 10^5^ cells/well) were seeded into each well of 24-well plates and added with TNF-α (10 ng/mL) and IFN-γ (10 ng/mL) in the presence or absence of FSE (25, 50, 100, 200, and 400 μg/mL). Dexamethasone (10^−4^ M) was used as a positive control. The TARC, MDC, and RANTES protein levels were determined by ELISA after 24 h of cultivation. Results are expressed as mean ± S.E.M. of three independent experiments. ^###^ *p* < 0.001 versus Normal (untreated group) * *p* < 0.05, ** *p* < 0.01, and *** *p* < 0.001 versus Control (TNF-α and IFN-γ-treated group).

Keratinocytes are known to participate in the cutaneous immune response in AD [25]. Thus, we examined the effect of FSE on the expression of chemokines in TNF-α and IFN-γ-stimulated HaCaT keratinocytes. We first tested the cytotoxicity of FSE using an MTT assay. The cells were exposed to various concentrations of FSE for 24 h. Dexamethasone was used as a positive control. FSE did not affect cell viability in keratinocytes (Fig 6A). To investigate the effect of FSE on the chemokines, HaCaT cells were stimulated with TNF-α/IFN-γ in the presence or absence of FSE for 24 h. FSE inhibited TNF-α/IFN-γ-induced production of TARC, MDC, and RANTES in HaCaT cells (Fig 6B, 6C and 6D). These results suggest that FSE restored the inflammatory response by down-regulating the chemokines in AD-like skin lesions.

### HPLC analysis of FSE reveals that FSE contains the four main chemical constituents (forsythiaside, phillyrin, pinoresinol, and phylligenin)

**Fig 7:**
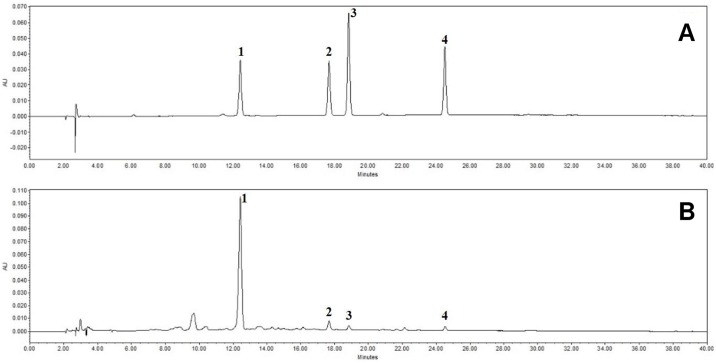
HPLC analysis of FSE reveals that FSE contains four main chemical constituents. HPLC chromatogram of a standard mixture (A) and *Forsythia suspensa* extract (B) at 235 nm. Forsythiaside (1), phillyrin (2), pinoresinol (3), and phylligenin (4) appeared with retention times of approximately 12.5 min, 17.7 min, 18.8 min, and 24.5 min, respectively.

The HPLC analysis of FSE revealed four peaks matching those of the commercial standards forsythiaside, phillyrin, pinoresinol, and phylligenin with retention times of approximately 12.5 min, 17.7 min, 18.8 min, and 24.5 min, respectively (Fig 7). The FSE contained 5.996 ± 0.007 mg/g forsythiaside, 0.422 ± 0.001 mg/g phillyrin, 0.159 ± 0.002 mg/g pinoresinol, and 0.136 ± 0.000 mg/g phylligenin (Table 2).

**Table 2:** Average contents of the reference compounds in *Forsythia suspensa* extract (*n* = 3).

Compound	Average content (mg/g)
Forsythiaside	5.996 ± 0.007
Phillyrin	0.422 ± 0.001
Pinoresinol	0.159 ± 0.002
Phylligenin	0.136 ± 0.000

### Forsythiaside, phillyrin, pinoresinol, and phylligenin inhibit the production of chemokines in human keratinocytes

**Fig 8:**
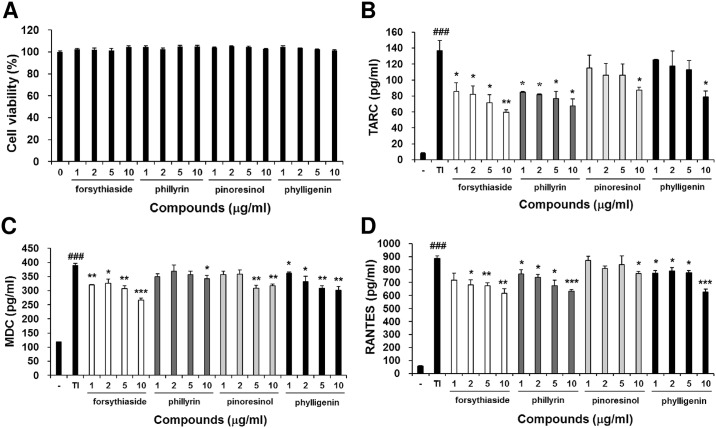
Forsythiaside, phillyrin, pinoresinol, and phylligenin inhibits the production of chemokines in TNF-α-and IFN-γ-treated human keratinocytes. (A) Cell viability measured using the MTT assay. HaCaT cells (3 x 10^5^ cells/well) were seeded into each well of 96-well plates and stimulated with or without compound (1, 5, 10, and 20 μg/mL) for 24 h. Production levels of TNF-α-and IFN-γ-induced (B) TARC, (C) MDC, (D) RANTES chemokines. HaCaT cells (3 x 10^5^ cells/well) were seeded into each well of 24-well plates and added with TNF-α (10 ng/ml) and IFN-γ (10 ng/mL) in the presence or absence of compound (1, 5, 10, and 20 μg/mL). The TARC, MDC, and RANTES protein levels were determined by ELISA after 24 h of cultivation. Results are expressed as mean ± S.E.M. of three independent experiments. ^###^ *p* < 0.001 versus Normal (untreated group) * *p* < 0.05, ** *p* < 0.01, and *** *p* < 0.001 versus Control (TNF-α and IFN-γ-treated group).

To determine the active components of FSE, we investigated the effect of forsythiaside, phillyrin, pinoresinol, and phylligenin on TNF-α/IFN-γ-induced chemokines production underlying the anti-allergic activity of FSE. In an MTT assay, the treatment of these compounds for 24 h had no significant cytotoxic effect on HaCaT cells (Fig 8A). Forsythiaside, phillyrin, pinoresinol, and phylligenin inhibited TNF-α/IFN-γ-induced production of TARC, MDC, and RANTES in a dose-dependent manner (Fig 8B, 8C and 8D). These results indicate that the inhibition effect of FSE in TNF-α/IFN-γ-induced chemokines production is due to the anti-allergic activity of forsythiaside, phillyrin, pinoresinol, and phylligenin.

## Discussion

Topical application of FSE in NC/Nga mice with DfE-induced AD ameliorated typical and histological changes of AD, such as dermatitis severity score, ear thickness, epidermal hyperplasia, and infiltration of inflammatory cells. Mast cells play a key role in allergic disorders through the release of a wide variety of products, including proteases, lipid mediators, histamine, cytokines, chemokines, and growth factors [26–28]. Histamine, a marker for mast cell degranulation, causes vasodilation, increased vascular permeability, and, consequently, recruitment of leukocytes in the acute allergic response of AD [29]. Pro-inflammatory cytokines, such as TNF-α and IL-4, initiate the chronic response of AD via enhancement of T cell activation or B cell survival [30]. After antigen presenting, B cells-produced antigen-specific IgE binds to FcεRI receptors on the mast cell surface, and activated mast cells release allergic mediators to induce allergic inflammation [23]. In AD mice, topical application of FSE reduced the number of mast cells in the skin lesions and the levels of histamine, TNF-α, and IgE in the serum. These results suggest that FSE suppresses allergic inflammation via inhibition of mast cell degranulation, especially the release of histamine. Although elevated serum IgE levels are a typical marker of AD, tacrolimus failed to reduce the serum IgE levels. Similarly, previous studies showed that tacrolimus did not inhibit the elevation of serum IgE levels in mice, although it suppressed the expression of IL-4 mRNA in the skin lesion [19, 31]. These results suggest that locally expressed IL-4 does not seem to contribute to the systemic IgE production.

Dysregulation of chemokines, cytokines, and adhesion molecules generally enhances the infiltration of immune cells into the inflammation site in the skin [32]. Th2 chemokines such as TARC and MDC play major roles in the pathogenesis of Th2-dominant skin disorders such as AD [33]. Previous studies have demonstrated that elevated serum levels of TARC and MDC correlate with skin disease severity in AD patients [34], and expression of TARC and MDC is increased in the lesional skin of AD patients [35]. RANTES secreted by keratinocytes recruits type 1 T cells or macrophages into skin lesions of AD and is involved in the infiltration and degranulation of eosinophils in the early stage of AD [36]. Adhesion molecules, such as ICAM-1 and VCAM-1, are expressed on keratinocytes, antigen-presenting cells, and endothelial cells, and are known to be involved in infiltration and activation of leukocytes into inflammation sites [37]. The expression of various adhesion molecules, chemokines, and cytokines on human keratinocytes is induced by pro-inflammatory cytokines such as TNF-α and IFN-γ [38–39]. A previous study showed that VCAM-1 blockade suppressed disease severity and inflammatory cells in an AD model [40]. Accordingly, down-regulation of these chemokines and adhesion molecules could be an effective target for the therapy of AD. Thus, we examined the ability of FSE on the expression of TARC, MDC, RANTES, TNF-α, IL-4, VCAM-1, and ICAM-1 molecules in the lesional skin and found that the expression levels of these proteins suppressed by FSE treatment. In addition, production of TNF-α/IFN-γ-induced TARC, MDC, and RANTES in HaCaT keratinocytes was effectively suppressed in a dose-dependent manner by FSE treatment. These findings suggest that FSE suppresses the expression of chemokines (TARC, MDC, and RANTES), cytokines (TNF-α and IL-4), and adhesion molecules (VCAM-1 and ICAM-1), thereby inhibiting migration and infiltration of leukocyte to sites of inflammation.

We found 4 main components in FSE which were forsythiaside, phillyrin, pinoresinol, and phylligenin. This study showed that these constituents decreased TNF-α/IFN-γ-induced production of TARC, MDC, and RANTES in HaCaT cells. Previous studies reported that these constituents had anti-inflammatory effects [41–44]. These results suggest that forsythiaside, phillyrin, pinoresinol, and phylligenin are the active compounds of FSE. Thus, even though anti-allergic effect of these compounds on AD was not elucidated yet, we assumed that these components might be responsible for the inhibitory effects of FSE on AD-like skin lesions.

## Conclusions

Topical application of FSE on lesional skin of DfE-induced AD mice effectively alleviated the development of AD-like lesions by suppressing the expression of chemokines, cytokines, and adhesion molecules in keratinocytes. Besides, among the components of FSE, forsythiaside, phillyrin, pinoresinol, and phylligenin inhibited the production of TARC, MDC, and RANTES in human keratinocytes. These results suggest that *F*. *suspensa* might be a useful candidate for treatment of allergic skin inflammatory disorders.
